# Attentional shifts by gaze direction in voluntary orienting: evidence from a microsaccade study

**DOI:** 10.1007/s00221-012-3260-z

**Published:** 2012-09-23

**Authors:** Takemasa Yokoyama, Yasuki Noguchi, Shinichi Kita

**Affiliations:** 1 Department of Psychology, Kobe University, 1-1 Rokkodai-cho, Nada-ku, Kobe, 657-8501 Japan; 2 Japan Society for the Promotion of Science, Tokyo, Japan

**Keywords:** Gaze direction, Attentional shift, Microsaccades, Voluntary orienting

## Abstract

Shifts in spatial attention can be induced by the gaze direction of another. However, it is unclear whether gaze direction influences the allocation of attention by reflexive or voluntary orienting. The present study was designed to examine which type of attentional orienting is elicited by gaze direction. We conducted two experiments to answer this question. In Experiment 1, we used a modified Posner paradigm with gaze cues and measured microsaccades to index the allocation of attention. We found that microsaccade direction followed cue direction between 200 and 400 ms after gaze cues were presented. This is consistent with the latencies observed in other microsaccade studies in which voluntary orienting is manipulated, suggesting that gaze direction elicits voluntary orienting. However, Experiment 1 did not separate voluntary and reflexive orienting directionally, so in Experiment 2, we used an anticue task in which cue direction (direction to allocate attention) was the opposite of gaze direction (direction of gaze in depicted face). The results in Experiment 2 were consistent with those from Experiment 1. Microsaccade direction followed the cue direction, not gaze direction. Taken together, these results indicate that the shift in spatial attention elicited by gaze direction is voluntary orienting.

## Introduction

The gaze direction of others is a rich source of social information. Being able to perceive and recognize another’s gaze is critical to constructing good social relationships. Recognition of gaze direction also plays an important role in predicting behavior, because gaze direction signals one’s purpose (Baron-Cohen 1995; Emery 2000). Therefore, the gaze direction of others influences the spatial attention of gaze perceivers (Friesen and Kingstone 1998, 2003; Itier et al. 2007; Langton et al. 2000; Ristic et al. 2002; Senju et al. 2008; Vecera and Rizzo 2006; Yokoyama et al. 2011).

Although numerous studies have found that gaze cues direct spatial attention, it is an open question whether this phenomenon is induced by reflexive or voluntary orienting. Reflexive orienting has an automatic character, whereas voluntary orienting is controlled. (Jonides 1981; Jonides and Yantis 1988; Muller and Rabbitt 1989; Yantis 1998). Typically, peripheral cues are used to induce reflexive orienting, and central cues are used to induce voluntary orienting. Although peripheral cues do not reliably predict target location, they trigger reflexive attention, so peripheral cues cannot be ignored (Remington et al. 1992; Yantis 1998). In contrast, symbolic cues can be ignored, so although central cues indicate left (for example), participants can ignore the meaning of the cues (Jonides 1981; Muller and Rabbitt 1989). Another important difference between them is the time taken to elicit attention. In reflexive attention, attentional shifts to peripheral targets occur instantaneously (Jonides 1981; Muller and Rabbitt 1989). On the other hand, in voluntary attention, attentional shifts triggered by central cues occur more slowly, because observers need to interpret the meaning of cues (Jonides 1981; Muller and Rabbitt 1989). In gaze cuing paradigms, although gaze cues are used as central cues that typically induce voluntary attention, some studies suggest that gaze cues trigger reflexive attention, while others suggest that they trigger voluntary attention.

Previous research supporting reflexive orienting as the trigger for gaze-directed attentional shifts has shown that responses to a peripheral target are faster when gaze direction, rather than other cues such as arrows or words, is used as a central cue (Driver et al. 1999; Friesen and Kingstone 1998, 2003; Ristic et al. 2002). However, this effect only occurs when stimulus onset asynchrony (SOA) is short, such as 100 ms. In this sense, such studies virtually guarantee that shifts of spatial attention elicited by gaze direction are reflexive. In contrast, other research suggests that gaze direction cues direct attention in a voluntary manner (Itier et al. 2007; Vecera and Rizzo 2006). In a case study, Vecera and Rizzo (2006) examined a patient with frontal lobe damage whose voluntary control of cognitive and attentional processes was impaired, but his reflexive attentional processing was intact. This patient did well when peripheral cues were presented, but his performance was impaired when central gaze and word cues were presented. Vecera and Rizzo concluded that shifts in spatial attention toward the direction of gaze are triggered by voluntary orienting. Given these two lines of research, it is unclear which attentional process is related to gaze-directed shifts in spatial attention.

The purpose of this paper is to examine whether shifts in spatial attention elicited by gaze direction are triggered by reflexive or voluntary orienting. To answer this question, we utilize microsaccades as an index of attentional allocation. Microsaccades are miniature eye movements that occur when our eyes fixate, and thus they are categorized as fixational eye movements (Martinez-Conde et al. 2009; Pastukhov and Braun 2010; Rolfs 2009). Microsaccades are an important part of the human visual system, because they allow us to maintain the visibility of fixated objects (Martinez-Conde et al. 2006). In addition, microsaccades contribute to covert attention, and the direction of microsaccades indicates where an individual is directing attention (Engbert 2006; Engbert and Kliegl 2003; Galfano et al. 2004; Hafed and Clark 2002; Laubrock et al. 2010; Pastukhov and Braun 2010). When peripheral cues are shown, the relative frequency of microsaccades in the cue direction increases rapidly (Hafed and Clark 2002; Laubrock et al. 2005). In contrast, when central cues are shown, it takes more time for the relative frequency of microsaccades to move in the cue direction (Engbert and Kliegl 2003; Laubrock et al. 2008). These studies are consistent with research on attentional orienting, which indicates that reflexive orienting occurs rapidly and voluntary orienting occurs slowly. Therefore, we will build on the results of these studies to examine whether shifts in spatial attention elicited by gaze direction are reflexive or voluntary in nature.

The present study examined which type of attentional orienting is involved in shifts of spatial attention toward gaze direction by measuring the direction of microsaccades. In Experiment 1, we used an attentional cuing paradigm with gaze cues and measured microsaccades during the task. Gaze cues positioned in the center of the screen indicated target locations with a high probability. In Experiment 2, we used an anticue task to separate reflexive and voluntary orienting directionally. Thus, unlike Experiment 1, gaze direction and cue direction were different in Experiment 2—targets appeared opposite the direction of gaze with a high probability.

## Experiment 1

### Methods

#### Participants

Ten participants were recruited from the Psychology Department of Kobe University. Each participant gave informed consent after the nature of the study had been explained. All participants had normal or corrected-to-normal visual acuity, and all were naïve to the purposes of this experiment. Approval for the experiment was obtained from the ethics committee of the Department of Psychology, Kobe University, Kobe, Japan.

#### Apparatus

Eye movements were recorded with the EyeLink CL 1000 Desktop System (SR Research, Toronto, Canada) with a sampling rate of 500 Hz. Stimuli were displayed on a Dell Trinitron 14.1-inch CRT display with a resolution of 1,024 × 768 pixels. Displays and data collection were controlled with MATLAB, using the Psychophysics (Brainard 1997; Pelli 1997) and EyeLink toolboxes (Cornelissen et al. 2002), running on a Dell Precision PWS479 computer under Microsoft Windows XP (refresh rate was 60 Hz).

#### Stimulus

**Fig. 1:**
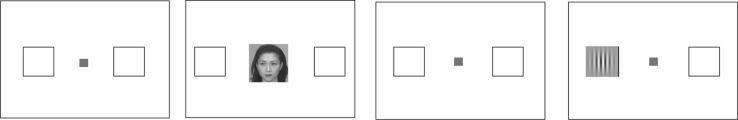
An example of the sequence of events for a typical trial. The first fixation display appeared for 500 ms, and then a central cue was presented for 100 ms. The second fixation display then appeared for 1,500–2,000 ms, and a target Gabor appeared for 200 ms

A red square (0.2° × 0.2°) was presented in the center of a uniform black background, and two placeholders (6.2° × 6.2°) were presented at 9.4° right/left of the center of the screen (Fig. 1). A Gabor patch (1° × 1°; spatial frequency: 0.143 deg/cycle) was used as a target and appeared in a white square frame. The direction of Gabor was vertical or horizontal, and luminance levels and root mean square (RMS) contrast levels were 14.7 and 8.9 cd/m^2^, respectively. We measured luminance and RMS contrast with a luminance and color meter (Konica Minolta CS-100A). For central gaze cues, we used six pictures (three male and three female; 6.2° × 6.2°) from the ATR DB99 database (ATR-Promotions, Kyoto, Japan). All faces had neutral expressions, and gaze direction was leftward, straight, and rightward. The rightward-gaze pictures were the mirror-reversed images of the leftward-gaze pictures. The overall luminance and contrast levels of the pictures were adjusted with Adobe Photoshop 6.0. After controlling luminance levels and RMS contrast, we conducted an analysis of variance (ANOVA) that indicated no significant differences between straight-gaze pictures and rightward-/leftward-gaze pictures for luminance levels (*F*_1,10_ = 0.000, *p* = 0.9969, n.s.) or RMS contrast levels (*F*_1,10_ = 0.000, *p* = 0.7668, n.s.). The means and standard errors of luminance level and RMS contrast are presented in Table 1.

**Table 1:** Mean and SD of luminance and RMS contrast of gaze cues (cd/m^2^)

	Front		Left/right	
	Mean	SD	Mean	SD
Luminance	17.789	3.773	17.779	3.562
Contrast (RMS)	10.559	1.340	10.307	1.283

#### Design and procedure

This experiment was a one-factorial repeated-measures design, with three levels of cue type: valid, invalid, and neutral. Cue direction was the same as gaze direction for this experiment; thus, the valid condition occurred when a target appeared at the placeholder indicated by the gaze direction, whereas in the invalid condition, the target appeared opposite the gaze direction. In the neutral condition, gaze direction was straight, and thus gaze direction did not indicate target location. Participants were tested individually. Each participant sat in a dark room with his/her chin in a chin rest, approximately 57 cm from a CRT screen.

Six experimental blocks of 36 trials were conducted, with 66.7 % of the trials in the valid condition, 16.7 % of the trials in the invalid condition, and 16.7 % of the trials in the neutral condition. When gaze direction was rightward or leftward, the target appeared at a placeholder indicated by gaze direction 80 % of the time, and participants were apprised of this probability beforehand. The experimental trials were preceded by 36 practice trials. Feedback was not given in either the practice or experimental trials.

A nine-point calibration procedure was conducted to align eye and screen coordinate systems before the start of every block. Drift correction was performed after every ninth trial. During a trial, if the participant’s gaze left a square with a side length of 2° of visual angle centered on the fixation, the trial would be automatically discarded, and the discarded trials were repeated in random order after the block finished.

Each trial cycled through the fixation display with two placeholders (500 ms), followed by a human face (cue) presented for 100 ms, whose gaze direction predicted the location of an upcoming target Gabor. Then, the fixation display with two placeholders was displayed again for a variable interstimulus interval selected at random from a uniform distribution of 1,500–2,000 ms. After that, a Gabor patch was displayed for 200 ms, and participants performed a two-alternative forced-choice orientation discrimination task. When the direction of the Gabor patch was vertical, they pressed “1”, and when the direction of the patch was horizontal, they pressed “2”.

#### Data analysis

Microsaccades were examined binocularly, and the data were acquired while the second fixation display was presented. Trials in which eye positions were more than 2° from the center of the screen and those in which eye blinks occurred during data acquisition were aborted.

**Fig. 2:**
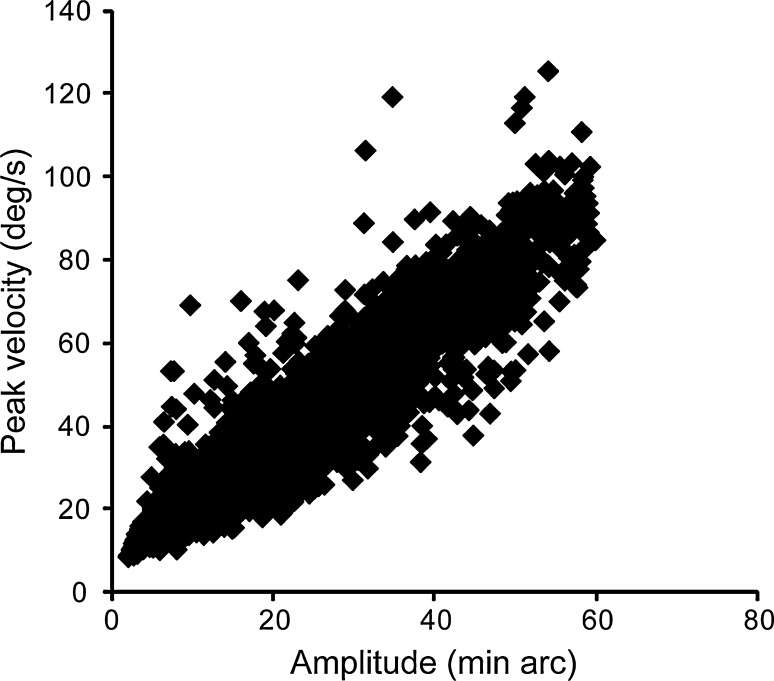
Peak velocities of microsaccades as a function of their amplitude. This plot has 5,970 microsaccades from 10 participants in Experiment 1

We used a modified version of the algorithm reported in Engbert and Kliegl (2003) to detect binocular microsaccades, adapted to the 500-Hz sampling rate used here. First, we transformed eye position data to velocities using a moving average of five data samples (10 ms) for each eye. Second, we computed the median-based standard deviation estimator as the velocity threshold and multiplied it by the relative velocity threshold (6.0). If the average velocity exceeded the velocity threshold in at least three consequent samples, we defined it as monocular microsaccades. Third, we defined binocular microsaccades when microsaccades occurred in both right and left eyes with a temporal overlap. Thus, binocular microsaccades were defined as microsaccades in this study. The microsaccades we extracted from the algorithm showed a strong correlation between peak velocity and amplitude (*r* = 0.947, *p* < 0.001), and thus use of this algorithm to extract microsaccades is reliable and valid in this study (Fig. 2). Microsaccades with amplitudes exceeding 1° were excluded from further analysis.

To analyze microsaccade direction, we first transformed microsaccade direction into polar coordinates. Histograms with a bin width of 30° were computed for time segments corresponding to periods of microsaccade-rate modulation. Thus, if the angle of a microsaccade’s direction was 12°, this direction would be defined as 0°. Rightward microsaccades were defined as being between 45° and 315°, and leftward microsaccades were defined as being between 135° and 225°. Upward (between 45° and 135°) and downward (between 225° and 315°) microsaccades were excluded from further analysis. We then classified microsaccades according to the direction of the cue and the direction of the horizontal and vertical component, but regardless of the valid and invalid conditions. After those microsaccades were classified into three postcue time windows (0–200 ms, 200–400 ms, and 400–600 ms; 0 ms equals the stimulus onset), we analyzed how frequently microsaccades oriented in the cue direction occurred in each time window. We analyzed microsaccades extracted in the neutral condition separately because attention was not manipulated in the neutral condition.

### Results

#### Reaction times

**Fig. 3:**
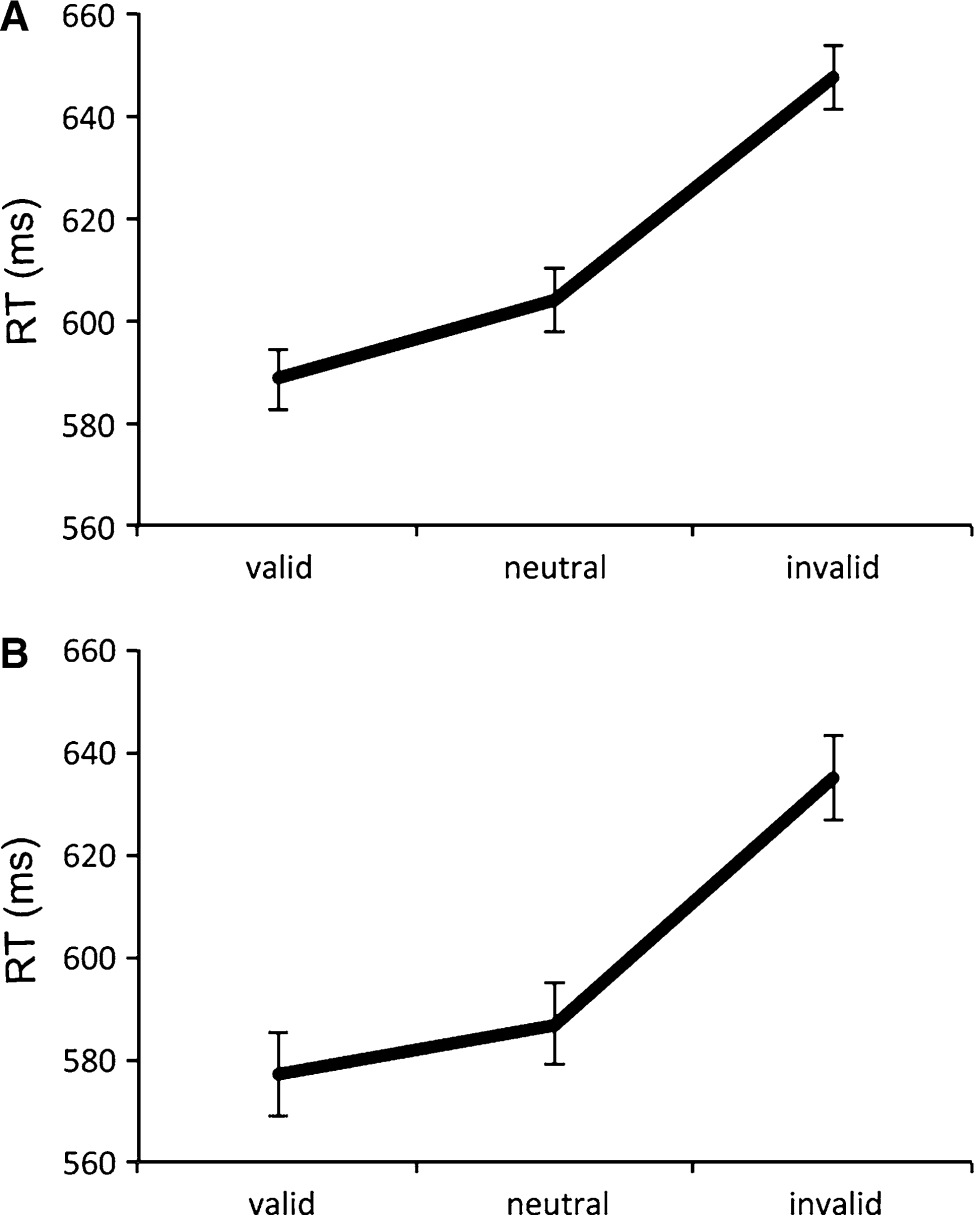
Mean reaction times in Experiment 1 (**a**) and 2 (**b**). The *error bars* represent standard error of mean, independent of between-subject variance (Loftus and Masson 1994)

Figure 3a shows reaction times for the three cue types. A one-factorial repeated-measures ANOVA of the reaction times found a significant main effect of cue type (*F*_2,18_ = 24.657, *p* < 0.001). A post hoc comparison with the Bonferroni correction confirmed a significant difference between the valid and invalid conditions (corrected *p* < 0.001) and between the neutral and invalid conditions (corrected *p* < 0.001). These results indicate that cue type facilitated reaction time, and therefore, we can infer effects for the attentional manipulation in Experiment 1.

#### Microsaccade

Before analyzing the relationship between cue direction and microsaccade direction in the valid and invalid conditions, we conducted a repeated-measures ANOVA of the frequency of left/right microsaccades in the neutral condition in each time window: 0–200 ms, 200–400 ms, and 400–600 ms. There were no significant differences in microsaccade direction in the 0–200 ms (*F*_1,19_ = 0.310, *p* = 0.591), 200–400 ms (*F*_1,19_ = 2.742, *p* = 0.13), and 400–600 ms (*F*_1,19_ = 0.000, *p* = 1.000) windows. Therefore, we confirmed that microsaccades were distributed almost equally leftward and rightward when attention was not manipulated.

**Fig. 4:**
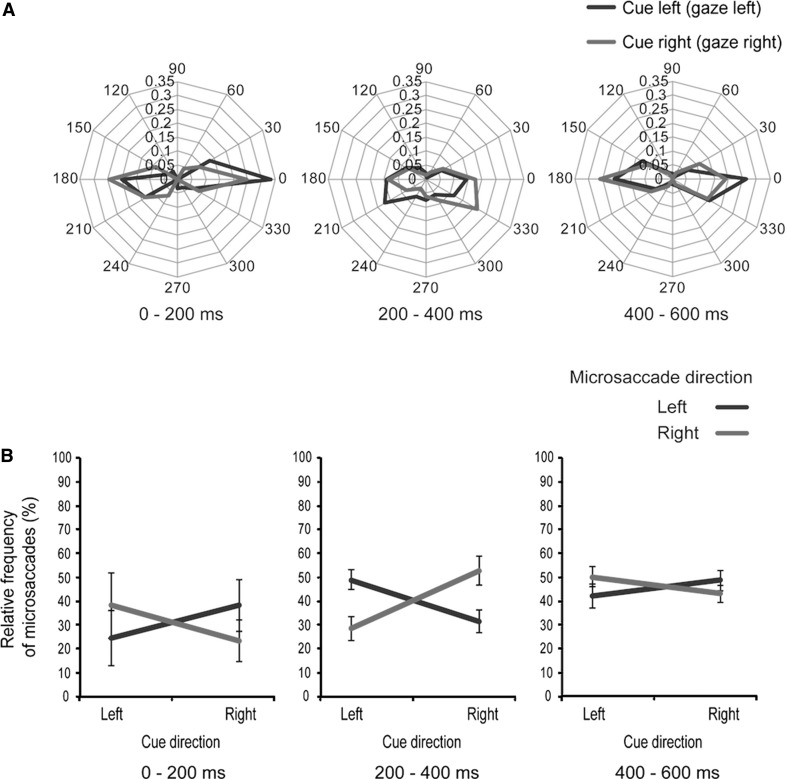
Microsaccade data in Experiment 1. **a** Polar plot histogram of microsaccades. Microsaccade directions in the three postcue time windows of Experiment 1: 0–200 ms, 200–400 ms, and 400–600 ms. The relative frequency of microsaccades is plotted in the *histograms* for *left* (*blue line*) and *right* (*red line*) cue directions. The *numbers on the outside of the histogram* indicate angle, and those on the *inside* indicate relative frequency of microsaccades. **b** Relative frequency of microsaccades. The *vertical axis* indicates the relative frequency of microsaccades, and the *horizontal axis* indicates cue directions. The *lines* indicate the relative frequencies of microsaccades to the *left* (*blue*) and the *right* (*red*). The *error bars* represent standard error of mean. For downward microsaccades in the 200–400 ms time window, a fixation was positioned below the *eyes of facial images*, so the differences in position between them might induce downward eye movement of some subjects when the screen switched from a facial image to fixation

Figure 4a shows polar plot histograms for the three postcue time windows: 0–200 ms, 200–400 ms, and 400–600 ms. We conducted a 2 (left/right cue direction) × 2 (left/right microsaccade direction) repeated-measures ANOVA of the frequency of microsaccades in each time window (Fig. 4b). In the 0–200 ms time window, the cue direction (*F*_1,39_ = 0.090, *p* = 0.770) and microsaccade direction (*F*_1,39_ = 0.085, *p* = 0.776) main effects were nonsignificant, as was the interaction (*F*_1,39_ = 0.988, *p* = 0.346). The main effects in the 200–400 ms time window were also nonsignificant (cue direction *F*_1,39_ = 1.879, *p* = 0.203; microsaccade direction *F*_1,39_ = 0.004, *p* = 0.9506), but we found a significant interaction between cue direction and microsaccade direction (*F*_1,39_ = 13.867, *p* < 0.01). To further assess the interaction between cue direction and microsaccade direction, we conducted a simple main-effects analysis. There were significant differences between left and right microsaccades in both the left cue direction (*F*_1,18_ = 5.514, *p* < 0.05: right microsaccades < left microsaccades) and the right cue direction (*F*_1,18_ = 5.982, *p* < 0.05: left microsaccades < right microsaccades). In the 400–600 ms time window, the main effects were not significant (cue direction *F*_1,39_ = 0.088, *p* = 0.773; microsaccade direction *F*_1,39_ = 0.044, *p* = 0.838), and the interaction was not significant (*F*_1,39_ = 1.579, *p* = 0.240). Taken together, these results indicate that microsaccades in the 200–400 ms window were oriented in the cue direction, but those in the 0–200 ms and 400–600 ms windows were not.

### Discussion

In Experiment 1, microsaccades were oriented in the cue direction when they occurred between 200 and 400 ms after the cue was presented, but when they occurred earlier (0–200 ms) or later (400–600 ms), they were not related to cue direction. Previous research on the pattern of microsaccade direction for voluntary orienting indicates that microsaccades are oriented in the cue direction after 200 ms when voluntary orienting is manipulated (Engbert and Kliegl 2003). On the other hand, previous research on the pattern of microsaccade direction for reflexive orienting indicates that microsaccade direction follows cue direction in short latency, and then microsaccades go with the opposite direction in long latency (Galfano et al. 2004; Hafed and Clark 2002; Laubrock et al. 2005; Rolfs et al. 2004). In this sense, the results obtained in Experiment 1 support the idea that shifts in spatial attention elicited by gaze direction are triggered by voluntary orienting.

Although we have concluded that shifts in spatial attention induced by gaze direction were triggered by voluntary orienting, the task we used in Experiment 1 did not separate reflexive and voluntary orienting directionally. Cue direction and gaze direction were the same in Experiment 1. This manipulation might have produced the results in Experiment 1. To address this potential methodological problem in Experiment 1, we used an anticue task in Experiment 2. In the anticue task, a target appears opposite gaze direction with a high probability, allowing us to separate reflexive and voluntary orienting directionally. In the next experiment, cue direction (and target location) is opposite to gaze direction, which is the valid condition for the next experiment; thus, validity is interpreted with respect to target location, not gaze direction.

In Experiment 2, we examined whether shifts in spatial attention elicited by gaze direction are triggered by voluntary orienting using two measures: direction and time period. With respect to direction, if shifts in spatial attention induced by gaze direction are triggered by reflexive orienting, microsaccades should be oriented in the same direction as gaze, whereas if they are triggered by voluntary orienting microsaccades, they should be biased in the likely direction of target. With respect to time period, if shifts in spatial attention induced by gaze direction are triggered by reflexive orienting, microsaccades should move in the same direction as gaze within 200 ms after the cue presentation. In contrast, if shifts in spatial attention induced by gaze direction are triggered by voluntary orienting, microsaccades should be directed away from the direction of gaze (in the cue direction) later than 200 ms after the cue, and we should replicate the results in Experiment 1.

## Experiment 2

### Methods

The method in Experiment 2 was the same as in Experiment 1, except for the following details.

#### Participants

Eight participants were recruited from the Psychology Department of Kobe University. Each participant gave informed consent after the nature of the study had been explained. All participants had normal or corrected-to-normal visual acuity, and all were naïve to the purposes of this experiment. Approval for the experiment was obtained from the ethics committee of the Department of Psychology, Kobe University, Kobe, Japan.

#### Design and procedure

Unlike Experiment 1, cue direction and gaze direction were different in this experiment. Thus, in the valid condition, targets appeared opposite the gaze direction, and in the invalid condition, targets appeared at a placeholder indicated by the gaze.

### Results

#### Reaction times

Figure 3b shows the mean reaction times for the three cue types. A one-factorial repeated-measures ANOVA of reaction times found a significant main effect of cue type (*F*_2,14_ = 14.361, *p* < 0.001). A post hoc comparison with the Bonferroni correction confirmed a significant difference between the valid and invalid conditions (corrected *p* < 0.001), and between the neutral and invalid conditions (corrected *p* < 0.001). We could not observe differences between the valid and neutral condition but did observe differences between the valid and invalid conditions, so we can infer that participants paid attention to the cue direction.

#### Microsaccade

Before we analyzed the relationship between cue direction and microsaccade direction in the valid and invalid conditions, we conducted a repeated-measures ANOVA of the frequency of left/right microsaccades in each time window for the neutral condition: 0–200 ms, 200–400 ms, and 400–600 ms. There were no significant differences in microsaccade directions in the 0–200 ms (*F*_1,15_ = 0.000, *p* = 0.992), 200–400 ms (*F*_1,15_ = 1.243, *p* = 0.301), and 400–600 ms (*F*_1,15_ = 1.294, *p* = 0.297) windows. Therefore, we confirmed that microsaccades were almost equally distributed to the left and right when attention was not manipulated.

**Fig. 5:**
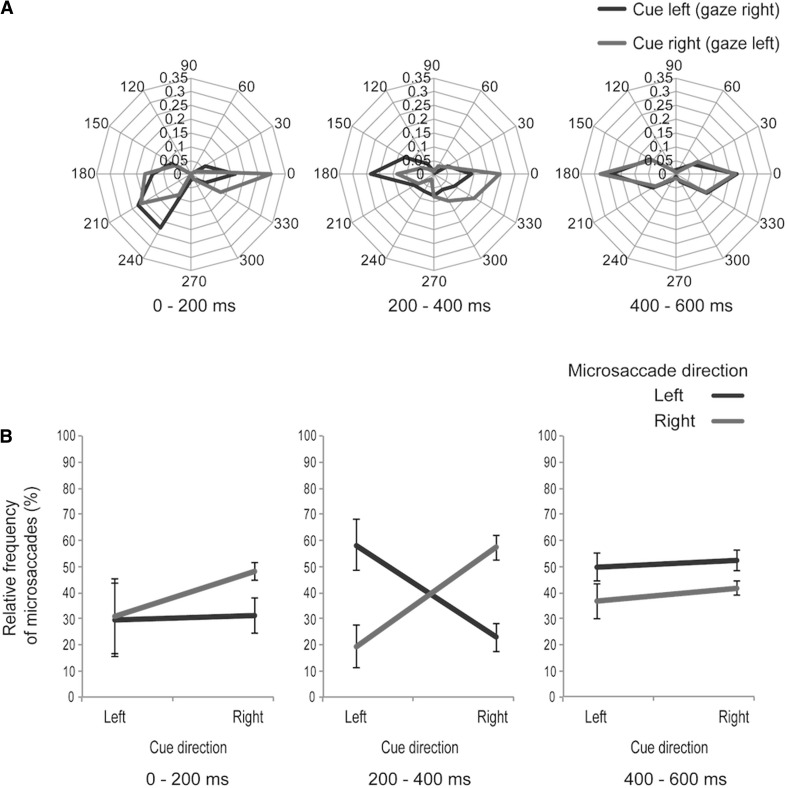
Microsaccade data in Experiment 2. Unlike Experiment 1, gaze direction and cue direction were different in Experiment 2. **a** Polar plot histogram of microsaccades. Microsaccade directions in the three postcue time windows of Experiment 1: 0–200 ms, 200–400 ms, and 400–600 ms. The relative frequency of microsaccades is plotted in the *histograms* for *left* (*blue line*) and *right* (*red line*) cue directions. The *numbers on the outside of the histogram* indicate angle, and those on the *inside* indicate relative frequency of microsaccades. **b** Relative frequency of microsaccades. The *vertical axis* indicates the relative frequency of microsaccades, and the *horizontal axis* indicates cue directions. The *lines* indicate the relative frequencies of microsaccades to the *left* (*blue*) and the *right* (*red*). The *error bars* represent standard error of mean

Figure 5a shows polar plot histograms of the three postcue time windows: 0–200 ms, 200–400 ms, and 400–600 ms. We conducted a 2 (left/right cue direction) × 2 (left/right microsaccade direction) repeated-measures ANOVA of the frequency of microsaccades in each time window (Fig. 5b). In the 0–200 ms time window, the main effects were not significant (cue direction *F*_1,31_ = 1.394, *p* = 0.276; microsaccade direction *F*_1,31_ = 0.504, *p* = 0.504), and the interaction was also nonsignificant (*F*_1,31_ = 0.960, *p* = 0.359). In the 200–400 ms time window, the main effects were not significant (cue direction *F*_1,31_ = 0.249, *p* = 0.633; microsaccade direction *F*_1,31_ = 0.111, *p* = 0.748), but the interaction between cue direction and microsaccade direction was significant (*F*_1,31_ = 12.472, *p* < 0.01). To assess this interaction further, we conducted a simple main-effects analysis. There were significant differences between left and right microsaccades in both the left (*F*_1,14_ = 9.923, *p* < 0.01: right microsaccades < left microsaccades) and right (*F*_1,14_ = 7.781, *p* < 0.05: left microsaccades < right microsaccades) cue directions. In the 400–600 ms time window, there were no significant main effects (cue direction *F*_1,31_ = 2.856, *p* = 0.134; microsaccade direction *F*_1,31_ = 1.906, *p* = 0.209), and the interaction was also nonsignificant (*F*_1,31_ = 0.225, *p* = 0.649). Taken together, these findings indicate that microsaccades were in the cue direction (opposite the gaze direction) in the 200–400 ms window, but not in the earlier (in 0–200 ms) or later (400–600 ms) windows.

### Discussion

In Experiment 2, we used an anticue task in which cue direction was different from gaze direction in order to confirm whether shifts in spatial attention induced by gaze direction are triggered by voluntary orienting. Microsaccades were in the cue direction (opposite to the gaze direction) in the middle time period, between 200 and 400 ms following the cue presentation. The response patterns in Experiment 2 are the same as those in Experiment 1, despite use of the anticue task, and they are also consistent with studies of voluntary orienting (Engbert and Kliegl 2003). Consequently, results in Experiment 2 indicate that shifts in spatial attention induced by gaze direction are triggered by voluntary orienting.

One might claim that our behavioral results are inconsistent with the study of Driver et al. (1999) using a similar anticue task, because faster reaction times (RTs) were observed in gaze direction, not direction of validity (opposition to gaze direction). However, this RT facilitation in their study was observed only when SOA was 300 ms, and faster RTs were observed in direction of validity when SOA was 700 ms. SOA in our study was between 1,500 and 2,000 ms, and therefore, our behavioral results and those of Driver et al. are consistent for long SOA.

Our data in Experiment 2 are not consistent with the results in the anticue task of Hafed and Clark (2002). Because their cue is a peripheral cue, and this manipulation usually elicits reflexive orienting, it is considered that microsaccade direction follows cue location, not direction suggested by cue validity (target location), in short latency. If attentional shifts elicited by gaze direction are reflexive orienting, the tendency of microsaccades in Experiment 2 should be similar to the result in the anticue task of Hafed and Clark (2002), but it is not. When previous microsaccade studies are taken together (Engbert and Kliegl 2003; Hafed and Clark 2002; Laubrock et al. 2005, 2008), the result in Experiment 2 is clearly indicating voluntary orienting, not reflexive orienting.

## General discussion

In this study, we investigated whether attentional orienting induced by the gaze direction of another human was reflexive or voluntary. We used gaze cues in an attentional cuing paradigm to examine this question, and we measured microsaccades as an index of attentional allocation. In Experiment 1, using standard gaze cues, microsaccades were only oriented in the cue direction between 200 and 400 ms after the cue presentation. This alignment of microsaccades in the cue direction in this particular time frame is consistent with results from previous studies of microsaccades in which voluntary orienting was manipulated, and thus, the results of Experiment 1 indicate that shifts in spatial attention directed toward gaze direction are triggered by voluntary orienting. The purpose of Experiment 2 was to separate reflexive and voluntary orienting directionally, to see whether the results obtained in Experiment 1 would persist. In Experiment 2, using an anticuing task, microsaccades were again oriented in the cue direction (despite being opposite to the gaze direction this time), and again this only occurred between 200 and 400 ms after cue presentation. In addition to replicating the results from Experiment 1, the results from Experiment 2 are also consistent with those from previous microsaccade studies. Taken together, these findings indicate that shifts in spatial attention directed toward gaze direction are triggered by voluntary orienting.

According to previous studies investigating the relationship between microsaccades and covert attention, the direction of microsaccades indicates the direction of covert attention (Engbert 2006; Engbert and Kliegl 2003; Hafed and Clark 2002; Laubrock et al. 2005, 2010; Rolfs 2009). Microsaccades were oriented in the cue directions only when we manipulated allocation of attention. This was especially impressive in Experiment 2—although the cue direction was the opposite of the gaze direction, microsaccades were oriented in the cue direction, not the gaze direction. Thus, our results are consistent with those from previous studies. Moreover, a previous study indicated that when reflexive attention is manipulated, the relative frequency of microsaccades in the cue direction increases rapidly, such as within 200 ms after stimulus onset (e.g., Laubrock et al. 2005). However, our data indicated that microsaccades were only oriented in the cue direction between 200 and 400 ms after cue onset. Thus, it is unlikely that the microsaccade effects we observed using gaze cues were triggered by reflexive orienting. Instead, considering both timing and directionality, our results clearly demonstrate that gaze cues elicit voluntary orienting.

Our results also support the learned association hypothesis proposed by Vecera and Rizzo (2006). In their case study, a patient with frontal lobe damage was unable to direct his attention based on centrally located gaze or word cues, but he could direct his attention based on peripheral cues. They proposed that orienting to another’s gaze was a learned association mechanism that relied on the frontal lobe. Our results, especially those from Experiment 2, are consistent with this idea. In Experiment 2, participants were required to orient away from the gaze direction to locate the target, and they were able to do this because they had “learned” the association between cue direction and target location, and that both were opposite to gaze direction.

Our results are inconsistent with studies indicating attentional shifts by gaze direction are reflexive in nature. This can be also explained by the learned association hypothesis. Gaze direction is repeatedly presented in real life, and gaze direction and its corresponding location are highly associated. Thus, gaze direction is rapidly reachable for inducing attentional shifts because gaze direction is an overlearned stimulus. However, attentional shifts by gaze direction are not reflexively induced. Farroni et al. (2000) investigated whether infants shifted their attention to gaze direction. They observed cuing effects caused by gaze direction only when the eyes of facial images moved from center to right or left, so they concluded that directional motion, not gaze per se, is necessary for inducing gaze cuing effects in infants. This means that attentional shifts by gaze direction are not innate, so attentional shifts by gaze direction should be caused by learning processes. Moreover, the study of Vecera and Rizzo (2006) indicates that a patient with frontal lobe damage could not direct his attention to the gaze direction although his reflexive attention caused by presentation of a peripheral cue was intact. His brain damage impaired learning processes (Vecera and Rizzo 2006), and hence this suggests that attentional shifts by gaze direction are induced in a voluntary, not reflexive, manner. Finally, the tendency of microsaccades in our results is totally different from the tendency of microsaccades that occurs when reflexive orienting is manipulated, whereas our results are consistent with studies of microsaccades where voluntary orienting is manipulated. Consequently, attentional shifts by gaze direction should be caused by the learning process and considered a voluntary process.

Although our data indicate a relationship between the direction of microsaccades and attention, a few studies do not support this idea. Horowitz et al. (2007) investigated whether microsaccades were causally related to reaction time for target detection, but they did not find facilitation of reaction time caused by microsaccades. Tse et al. (2002, 2004) used peripheral cues to examine the relationship between microsaccades and attention, but they did not observe microsaccades that were not oriented in the cue direction. Therefore, the relationship between the direction of microsaccades and attention is not perfectly verified; however, our study, at least, has clearly demonstrated a relationship between microsaccade direction and attentional direction.

## Conclusion

The purpose of this study was to examine which type of attentional orienting was involved in shifts of spatial attention toward gaze direction: reflexive or voluntary orienting. Our data indicated that this attentional process occurs via voluntary orienting, at least when microsaccades are used as an index of attention. Our experiments also demonstrated that microsaccades can be used to study the allocation of attention. Consequently, our results provide important information not only about shifts in spatial attention elicited by gaze direction but also about the effectiveness of measuring microsaccades as an indication of the allocation of attention.
